# The role of combined training timing strategies in mitigating the dawn phenomenon in older adults with type 2 diabetes in Nanjing, China: a randomized controlled trial

**DOI:** 10.1016/j.pmedr.2026.103456

**Published:** 2026-03-21

**Authors:** Yibo Gao, Xiang Pan, Yanfeng Zhang, Koya Suzuki, Mingzhe Li, Jin He, Lupei Jiang, Jie Li

**Affiliations:** a China Institute of Sport Science, Beijing 100061, China; b Graduate School of Health and Sports Science, Juntendo University, Inzai 270-1695, Japan; c Suojin Village Community Health Service Center, Nanjing 210042, China

**Keywords:** Dawn phenomenon, Periodic exercise, Older people, Autoregressive model

## Abstract

**Objective:**

To evaluate the effectiveness of a novel periodic exercise protocol on the dawn phenomenon in Chinese patients with type 2 diabetes and examine the impact of different inter-cycle rest days.

**Methods:**

93 participants in Nanjing, China (recruited from September 2023 to October 2023) were randomized into four groups for an intervention conducted from October 2023 to January 2024. Three intervention groups performed nine 5-day mini-cycles (90-min sessions of combined training) with inter-cycle rest days of 1, 2, or 3 days, respectively. Continuous glucose monitoring and lipid profiles were assessed pre- and post-intervention.

**Results:**

85 participants completed the study. All intervention groups showed significant improvements in glucose and lipid parameters (*P* < 0.05). Dawn phenomenon decreased below 1.11 mmol/L in all exercise groups, with the 1-day rest group showing the earliest and most pronounced improvements and a continuous decline from cycles 4 to 9.

**Conclusions:**

The protocol effectively improved the dawn phenomenon and metabolic outcomes. Shorter inter-cycle rest days produced more sustained benefits, suggesting that glycemic improvements are influenced by homeostatic regulation.

## Highlights

• Periodic exercise significantly reduced the dawn phenomenon in type 2 diabetes.
• Shorter inter-cycle rest days yielded earlier and sustained glucose improvements.
• All exercise groups showed significant glucose and lipid improvements over control.
• The 1-day rest group demonstrated the greatest dawn phenomenon reduction.
• Findings suggest a homeostatic ceiling effect in glucose fluctuation improvements.

## 1 Introduction

The number of people with diabetes mellitus has soared globally (Federation, 2025). The blood glucose status of patients can be roughly divided into three types, namely, hyperglycemia, hypoglycemia and blood glucose fluctuation (Hirsch, 2015). Abnormal fluctuation is very dangerous for type 2 diabetes patients (Peng et al., 2022). Schmidt et al. (1981) first described the dawn phenomenon in patients with diabetes. The dawn phenomenon is characterised by a spontaneous rise in blood glucose levels during the night-to-early-morning period. Furthermore, studies have indicated a strong correlation between glycemic variability and the development of both macrovascular and microvascular complications (Penno et al., 2013).

Exercise as an important intervention has been widely confirmed. Globally, various exercise modalities have gained popularity as effective strategies for managing cardiometabolic health in individuals with type 2 diabetes (Khalafi et al., 2025a; McAvoy et al., 2025). Current evidence strongly supports the role of these structured physical activities in improving diverse health-related markers (Al-Mhanna et al., 2025b). In particular, the effectiveness of combined training programs, which integrate both aerobic and resistance exercises—has been increasingly highlighted for yielding comprehensive benefits in glycemic control and overall cardiometabolic profiles (Al-Mhanna et al., 2025a; Al-Mhanna et al., 2025b). Furthermore, regular exercise can improve insulin sensitivity and blood lipid, reduce inflammation (Tongjian et al., 2013), regulate the level of oxidative stress (Laughlin et al., 1990), and thus play a positive role in diabetes management. However, there are only two studies on exercise intervention for dawn phenomenon at present. The study by Zheng et al. (2020) had 20 dawn phenomenon patients do 30 min of moderate-intensity aerobic exercise before breakfast. The results show that acute moderate-intensity aerobic exercise before breakfast can reduce the increase of blood glucose in the early morning of patients with type 2 diabetes and partially mitigate dawn phenomenon. Paing et al. (2019) conducted a 14-day exercise experiment on dawn phenomenon, and exercised for three minutes after every 60 min, 30 min and 15 min during seven hours of sedentary time every day. The results showed that the fasting blood glucose value and the variability of blood glucose at night in patients with an interval of 15 min were significantly higher than those in the other two groups, and the reduction in dawn phenomenon was also significantly more pronounced than that in the other two groups. However, both of these studies were limited by small sample sizes, short intervention periods, and a single outcome measure. Additionally, Su et al. (2023) conducted a systematic review and meta-analysis to investigate the effectiveness of resistance training with varying repetition counts in reducing hemoglobin A1c and fasting blood glucose levels among type 2 diabetes patients, confirming that the number of repetitions per set is a key factor in the effectiveness of reducing hemoglobin A1c and fasting blood glucose levels among type 2 diabetes patients.

The objective of this study was to investigate the dawn phenomenon in patients with type 2 diabetes by implementing a novel periodic exercise intervention and expanding the study population. Specifically, (1) validate the effectiveness of exercise in improving dawn phenomenon using continuous glucose monitoring, (2) implement a nine mini-cycle intervention (27 sessions over 7–10 weeks) to enable multi-level comparisons across time rest days, evaluate both blood glucose and lipid outcomes, (3) apply an autoregressive model to enhance dawn phenomenon detection and verification.

## 2 Methods

### 2.1 Study design and population

This study was designed as a single-blind with participants, parallel-group randomized controlled trial consisting of four arms: three exercise intervention groups and one control group. The primary aim was to evaluate the effects of different periodic exercise interventions on the dawn phenomenon in patients with type 2 diabetes. The intervention comprised nine mini-cycles (a total of 27 training sessions), completed over a period of 7–10 weeks.

Participants were older adults (60–75 years) diagnosed with the dawn phenomenon (≥ 1.11 mmol/L). Detailed inclusion and exclusion criteria are provided in Section 2 in the Supplementary Materials. Sample size was calculated using G*Power 3.1 with dawn phenomenon as the primary outcome (f = 0.25, α = 0.05, power = 0.80, correlation = 0.50), requiring ≥68 participants. Allowing 15.00% attrition, 20 per group were targeted. Participants were recruited from September to October 2023 at Suojin Village Community Health Service Center (Nanjing, China). The subsequent exercise intervention and data collection were conducted from October 2023 to January 2024. Participants provided informed consent and were randomized (1:1:1:1) via computer-generated sequences with sealed envelopes. Participants were blinded to allocation; assessors were not.

All participants first completed a 3-day adaptation Training. On Day 1, they were instructed in exercise techniques; on Day 2, they underwent intensity testing and the talk test; and on Day 3, they performed a formal familiarization session at 70% of the prescribed training intensity. Detailed exercise protocols are provided in Fig. S1. RCT CONSORT diagram see Fig. S2. 48 h after completing the adaptive training, volunteers underwent fasting measurements in the early morning, wore the continuous glucose monitoring system, were informed of the sensor replacement schedule, and then proceeded with the full exercise intervention protocol (Table S1). Furthermore, physicians judged the dosage of medication based on changes in indicators. All participants followed a standardized diet designed in accordance with dietary guidelines for diabetes management and were instructed to adhere strictly to the protocol (see Supplemental Content 2: Dietary Control Plan).

All exercise sessions were fully supervised by trained staff at seven designated community venues to ensure protocol compliance and participant safety. Adherence to the intervention was measured via mandatory attendance check-ins for every session. Similarly, dietary adherence was monitored through regular questionnaires to ensure participants followed the personalized nutritional recommendations and to account for potential habitual dietary confounding factors.

The 90-min exercise protocol followed American College of Sports Medicine and American Diabetes Association guidelines, comprising aerobic gymnastics and resistance training (two sets of 10 repetitions). Detailed movement standards and safety protocols are available in Tables S1 and S2. Based on evidence that the insulin-sensitizing effect of moderate exercise dissipates within 48 h and the principle of supercompensation (Kido et al., 2020). The intervention adopted a 5-day mini-cycle (exercise on Days 1, 3, and 5; rest on Days 2 and 4). Inter-cycle rest days varied among groups: 1 day (Group 1), 2 days (Group 2), or 3 days (Group 3). Each group completed nine mini-cycles (27 sessions in total) to ensure equivalent exercise volume. The control group received no intervention.

This study was conducted at the Suojin Village Community Health Service Center (Xuanwu District, Nanjing, China) and approved by the Ethics Committee of the China Institute of Sports Science (No. CISSLA-20221116). All procedures complied with the Declaration of Helsinki, Good Clinical Practice, and relevant Chinese regulations. Written informed consent was obtained from all participants. Data were collected by trained medical staff and kept strictly confidential for research purposes only. This study was conducted after a retrospective clinical enrollment on ClinicalTrials.gov (NO. NCT06936982; Date: 2025-04-20).

### 2.2 Measures

Fasting blood glucose and blood glucose were measured by continuous glucose monitoring testing system using Yuwell CT15 (Jiangsu Yuwell POCTech Biotechnology Co., Ltd., Jiangsu, China), which records glucose values every three minutes and can operate continuously for up to 14 days. In addition, capillary glucose levels (fasting, 2 h after-breakfast, and 2 h after -dinner) were obtained with the Yuwell 310 device for continuous glucose monitoring calibration. Confirming the consistency between continuous glucose monitoring and capillary glucose measurements (Section 5 in Supplementary Materials). Dawn phenomenon was calculated as the difference between the highest blood glucose level before breakfast and the lowest nocturnal blood glucose value during 0:00–6:00 AM. Dawn phenomenon = the highest blood glucose level before breakfast – the lowest blood glucose level at night.

Total cholesterol, triglycerides, high-density lipoprotein and low-density lipoprotein were measured using the AU5800 automatic biochemical analyzer (Beckman Coulter, Pasadena, USA). Hemoglobin A1c was measured from venous blood samples using a turbidimetric method. Given that the intervention lasted 9 sessions, hemoglobin A1c was considered a secondary outcome and used solely to monitor trends in glycemic control.

### 2.3 Statistical analysis

Shapiro-Wilk test was used to test the normal distribution of all data. If the data met the normal distribution, logarithms were taken as base 10 if they did not. All data presentation forms were expressed as mean ± standard deviation (X ± S). The paired sample T test was performed within the group, and one-way analysis of variance was used for between-group analysis. The standard deviation was also calculated using the Bland–Altman method. The standard error of measurement for lipid parameters was calculated using the formula standard error of measurement$=\mathrm{SD}/\sqrt{n}$ to determine whether observed changes exceeded the measurement error. Statistical significance was set at α = 0.05, and results with *p*-values <0.05 were considered statistically significant.

An autoregressive model was employed to analyze temporal changes in the effects of exercise across mini-cycles. The underlying assumption of this time series model is that linear relationships exist between successive measurements, rendering it well-suited for the analysis of continuous glucose monitoring data collected repeatedly over multiple cycles. By calculating the coefficients between adjacent mini-cycles, it is possible to determine whether each exercise cycle enhances or diminishes outcomes. In comparison with simple pre- and post-intervention, the autoregressive model has been shown to offer a more sensitive means of identifying fluctuation patterns and determining the most effective cycle frequency. SPSS 29.0 (IBM Corp., Armonk, NY, USA), AMOS 26.0 (IBM Corp., Armonk, NY, USA) and MedCalc 23.3.4 (MedCalc Software Ltd., Ostend, Belgium) were used for statistical analysis.

## 3 Results

### 3.1 Baseline

A total of 93 participants were enrolled, including 70 in the intervention groups and 23 in the control group. During the intervention, four participants (three men, one woman) withdrew; three modified their medications, and one engaged in additional exercise. Consequently, 85 participants (64 intervention, 21 control) completed the study and provided full data, resulting in an overall dropout rate of 8.60% (8/93). Furthermore, during the entire intervention period, no severe exercise-related adverse events were reported in any of the groups. None of the participants were using insulin. Details of other medications and dosing frequencies are presented in Table S2. Baseline characteristics were comparable across the four groups, with no significant differences observed (all *P* = 0.05–0.94; Table 1).

**Table 1:** Baseline physiological and biochemical characteristics of Chinese older adults with type 2 diabetes mellitus in Nanjing, China, collected in October 2023.

		Group 1 (n = 22)	Group 3 (n = 21)	Group 3 (n = 21)	Group 4 (n = 21)
Age (years)		68.24 ± 5.81	68.27 ± 5.44	67.63 ± 6.62	68.54 ± 6.15
Diabetes duration (years)		10.83 ± 6.38	10.92 ± 7.21	11.18 ± 6.32	11.42 ± 7.24
Gender	Male/Female	1.00	1.10	0.91	1.10
Lipids (mmol/L)	Total cholesterol	4.88 ± 0.90	5.01 ± 1.17	4.92 ± 0.89	4.94 ± 0.94
	Triglycerides	1.69 ± 1.01	1.70 ± 0.80	1.59 ± 0.93	1.64 ± 0.90
	High-density lipoprotein	1.41 ± 0.28	1.39 ± 0.38	1.40 ± 0.33	1.38 ± 0.31
	Low-density lipoprotein	2.97 ± 0.66	3.02 ± 0.75	3.00 ± 0.54	3.01 ± 0.72
Glucose metabolism(mmol/L)	Fasting blood glucose	8.61 ± 1.74	8.58 ± 1.68	8.59 ± 1.72	8.62 ± 1.70
	Hemoglobin A1c (%)	6.91 ± 1.28	6.88 ± 1.43	6.87 ± 1.37	6.90 ± 1.36
	dawn phenomenon	2.40 ± 1.24	2.35 ± 0.88	2.43 ± 1.07	2.34 ± 1.57

One-way ANOVA was used for between-group analysis. *, p < 0.05; **, p < 0.01.

The overall adherence rate to the exercise protocol was 92.50%, with no significant differences among the three intervention groups. Given the uniformly high adherence, a sub-analysis based on adherence levels was not deemed necessary.

### 3.2 After exercise intervention

**Fig. 1:**
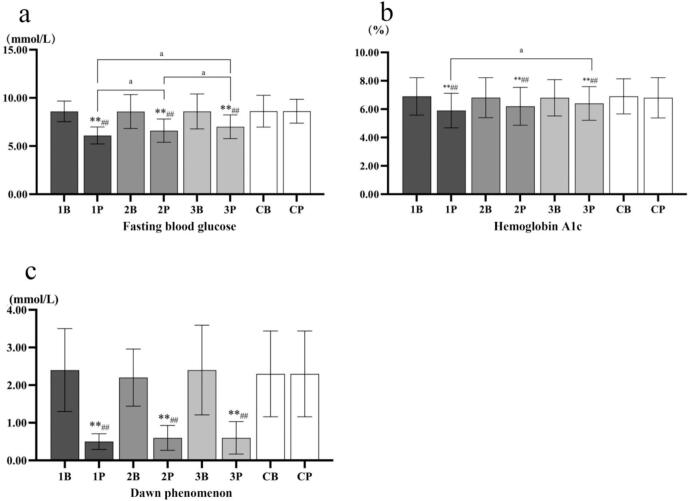
Changes in blood glucose indicators among Chinese older adults with type 2 diabetes in Nanjing, China, from October 2023 to January 2024. B, baseline; P, post-intervention. 1B/1P: Group 1; 2B/2P: Group 2; 3B/3P: Group 3; 4B/4P: Control group. The paired sample T test was performed within the group, and one-way analysis of variance was used for between-group analysis. * is compared to Baseline data, # is posttest compared to control, and a is compared to Group 1, Group 2, and Group 3. *, *p* < 0.05; **, *p* < 0.01; #, p < 0.05; ##, p < 0.01; a, p < 0.05; aa, *p* < 0.01.

In all three exercise intervention groups, the mean absolute changes in the four indicators exceeded their respective standard error of mean values, indicating that the observed improvements were greater than the measurement error (Table S3). In addition, among blood glucose indexes: the three indexes also showed the same rule in compared with baseline and control group (*P* < 0.01), while only fasting blood glucose showed significant difference between Group 1 and Group 2 and Group 3 (*P* < 0.05). See Fig. 1. Comprehensive statistical parameters, including 95% confidence intervals (CIs) and exact effect sizes for glucose metabolism indicators, are provided in Table S3. Notably, the primary outcome dawn phenomenon showed substantial improvement in Group 1 (mean change = −1.90 mmol/L, 95% CI [−2.43, −1.37], Cohen's *d* = 1.96), which was reported first as the primary outcome.

**Fig. 2:**
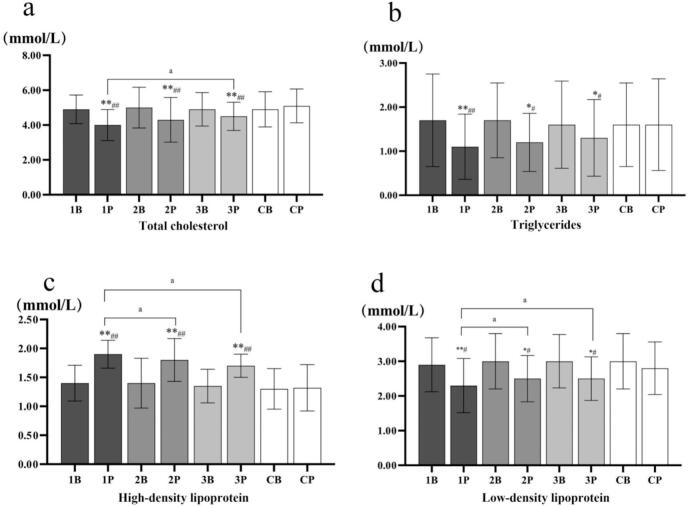
Changes in blood lipid profiles among Chinese older adults with type 2 diabetes in Nanjing, China, from October 2023 to January 2024. B, baseline; P, post-intervention. 1B/1P: Group 1; 2B/2P: Group 2; 3B/3P: Group 3; 4B/4P: Control group. The paired sample *t-*test was performed within the group, and one-way analysis of variance was used for between-group analysis. * is compared to Baseline data, # is posttest compared to control, and a is compared to Group 1, Group 2, and Group 3. *, p < 0.05; **, p < 0.01; #, p < 0.05; ##, p < 0.01; a, p < 0.05; aa, p < 0.01.

All four indicators showed significant differences both when compared with the baseline and with the control group (P < 0.05, total cholesterol: 1P vs 1B/CP, P < 0.01; 2P vs 2B/CP, P < 0.01; 3P vs 3B/CP, *P* < 0.01. triglycerides:1P vs 1B/CP, P < 0.01. high-density lipoprotein:1P vs 1B/CP, P < 0.01; 2P vs 2B/CP, P < 0.01; 3P vs 3B/CP, P < 0.01. low-density lipoprotein:1P vs 1B/CP, P < 0.01). In addition, there was a significant difference between the tail test of Group 1 and Group 3 in total cholesterol (P < 0.05), and there was also a significant difference between Group 1 and Group 2 and Group 3 in high-density lipoprotein (P < 0.05) (Fig. 2). Detailed mean changes, 95% CIs, and effect sizes for all lipid profiles are summarized in Table S3. For example, total cholesterol in Group 1 demonstrated a large effect size following the intervention (mean change = −0.90 mmol/L, 95% CI [−1.14, −0.66], Cohen's *d* = 1.05).

### 3.3 Effect on the dawn phenomenon

The autoregressive model was applied to quantify the stability of glucose fluctuations across cycles. A higher coefficient indicates a faster and more stable recovery of glucose homeostasis following the exercise intervention. The model structure was first tested for goodness-of-fit, through chi-square divided by degrees of freedom, root mean square error of approximation, goodness of fit index, comparative fit index and incremental fit index compared with the standard to determine. The model structure was tested for goodness-of-fit, and all autoregressive models for the three intervention groups demonstrated acceptable fit indices within the recommended guidelines (Table S4).

**Fig. 3:**
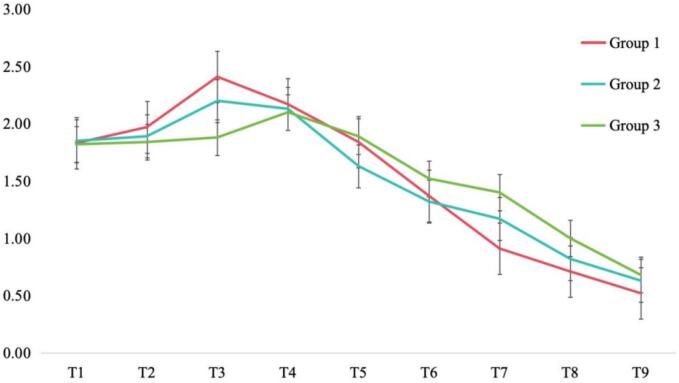
Absolute change values of the dawn phenomenon across nine mini-cycles among Chinese older adults with type 2 diabetes mellitus in Nanjing, China, from October 2023 to January 2024. (mmol/L). T1, T2......T9 represent the 1st and 2nd......9 Mini-cycles, respectively.

The dawn phenomenon change values of each group (Fig. 3) were combined and analyzed with the model regression coefficients (Table 2). The dawn phenomenon of Group 1, Group 2, and Group 3 was less than 1.11 mmol/L at T7, T8, and T9, respectively. Group 1 showed the largest change in the third cycle, and the wave peak of Group 1 was higher than that of Group 2 and Group 3(*P* < 0.01). Group 1 showed a decreasing trend from T4 to T9, and the decreasing trend of Group 1 was the largest, followed by Group 2. The autoregressive coefficients of dawn phenomenon in different cycles show that the regression coefficients of Group 1 suddenly increase in T3-T4, while that of Group 3 has little change in this cycle. Subsequently, the regression coefficient of Group 1 gradually decreases, while that of Group 3 is basically flat and then decreases in T4-T8. However, Group 2 showed the same pattern of change as that of Group 1, and the amount of change was not as large as that of Group 1 (Table 2).

**Table 2:** Regression coefficients of the dawn phenomenon between mini-cycles among older adults with type 2 diabetes mellitus in Nanjing, China from October 2023 to January 2024.

	Group 1	Group 2	Group 3	F	P
T1—T2	0.41	0.43	0.43	2.72	0.08
T2—T3	0.52	0.46	0.42	3.30	<0.05*
T3—T4	0.71	0.66	0.57	10.91	<0.01**
T4—T5	0.32	0.31	0.30	1.14	0.33
T5—T6	0.28	0.28	0.24	1.92	0.16
T6—T7	0.26	0.23	0.32	7.31	0.01**
T7—T8	0.15	0.14	0.28	3.90	0.03*
T8—T9	0.14	0.13	0.16	0.43	0.69

T1, T2......T9 represent the 1st and 2nd......9 Mini-cycles, respectively. *, p < 0.05; **, p < 0.01.

## 4 Discussions

Across all intervention groups (Group 1–Group 3), periodic exercise led to varying degrees of improvement from baseline, with Group 1 showing the most pronounced changes. Dawn phenomenon progressively declined in all groups, reaching <1.11 mmol/L earliest in Group 1. Equality of regression coefficients between periods in Group 1 and Group 2 indicated that, despite continued reductions, the rate of decline gradually slowed.

The mean dawn phenomenon after the periodical exercise was <1.11 mmol/L in all groups. In blood glucose fluctuation, this result is due to the short time interval (1 day) between Group 1 mini-cycles and mini-weeks, which allows us to achieve the effect of 1 day of exercise and 1 day of rest in continuity. The cumulative effect of the exercise intervention was greater than the debilitating effect, as Group 1 performed more physical activity during the same period.

The varying improvements in the dawn phenomenon across rest intervals stem from inconsistent recovery periods. Longer rest weakens the cumulative effect of exercise, creating a bottleneck in blood sugar reduction. From the recovery perspective, it is more similar to the principle of over-recovery (Ren et al., 1994). Additionally, the body's metabolism increases after exercise. The greater the intensity of exercise, the more energy is consumed. Recovery encompasses improved β-cell function, insulin sensitivity, and lipid metabolism, alongside restored anti-inflammatory and anti-oxidative stress enzymes. Prolonged exercise may induce a “de-compensation” in these factors, causing a “plateau phase” or slower decrease in Group 2 and 3. Group 2 and Group 3 have a “plateau phase”, but will still decline slowly (Nakatani et al., 1997), which indirectly indicates that the body's regulation of dawn phenomenon is becoming more and more accurate.

All measured outcomes demonstrated significant improvements (*p* < 0.05) both compared to pre-intervention values and the control group. Exercise can improve blood lipid and glucose metabolism indicators in diabetes patients (Kataoka et al., 2025), and a similar pattern is seen in dawn phenomenon patients. In this exercise protocol, we followed moderate aerobic intensity and resistance training. Studies conducted by Gavin et al. (2010) in the American continent, Okada et al. (2010) in the Asian continent, and Kahleova et al. (2011) in the European continent have demonstrated that all four indicators of lipid metabolism show effective improvements in the blood lipid metabolism. (Al-Mhanna et al., 2024) of the present study. Beyond regional studies, our findings strongly align with recent literature emphasizing the vital role of various structured exercise modalities in populations with metabolic dysregulation. Specifically, concurrent training has been consistently shown to optimize overall cardiometabolic health indices in individuals with type 2 diabetes (Khalafi et al., 2025b). Similarly, the implementation of continuous aerobic and interval training provides robust cardiovascular resilience and glycemic control (Al-Mhanna et al., 2025a). Furthermore, incorporating resistance training remains crucial for enhancing muscle-mediated glucose disposal and regulating lipid profiles (Batrakoulis et al., 2022). Comparing our present results with these established paradigms further validates the comprehensive metabolic benefits observed in our periodic combined training protocol. The improvements can be primarily attributed to exercise-induced enhancement of fat oxidation, which reduces the production of reactive oxygen species and alleviates the suppression of β-cell transcription (Pullen and Rutter, 2013). Additionally, exercise improves insulin resistance, thereby reducing hepatic lipid synthesis and preventing the overstimulation of β-cells caused by abnormal lipid metabolism. It also helps regulate lipid synthesis and storage, as well as modulate levels of related hormones (Shih et al., 2013), ultimately contributing to improved lipid metabolism.

Despite significant improvements, glucose levels did not normalize, likely due to the long diabetes mellitus duration (over 10 years) among participants. Chronic hyperglycemia is associated with impaired insulin secretion by pancreatic β cells, defective receptor phosphorylation, and reduced target cell sensitivity. These factors collectively limit the ability of exercise to restore normal glycemic (Kadowaki, 2000). Consistent with previous meta-analyses, our periodic exercise protocol significantly reduced hemoglobin A1c levels. This improvement results from increased glucose utilization during exercise, which reduces the rate of hemoglobin glycation.

Except for triglycerides and dawn phenomenon, significant differences in total cholesterol and hemoglobin A1c were observed primarily between Group 1 and Group 3 (*P* < 0.05). These results suggest that shorter inter-cycle rest days (Group 1) ensure that the cumulative effects of exercise are maintained, preventing key metabolic enzymes from returning to baseline. Meanwhile, appropriate intervals help reduce oxidative stress and inflammation caused by prolonged activity, thereby improving overall metabolic stability (Green et al., 2017).

The correlation coefficients were obtained by introducing the autoregressive model to process the blood glucose after 24 h of exercise during each week. The autoregressive model is one of the most commonly used models for dealing with time series. It adopts the assumption of the current value of the time series or calculates or predicts through a linear combination of its previous values, reflecting the real world in a relatively reliable theoretical framework (Parlak et al., 2022). Similar autoregression-based approaches have been used in related studies, including real-time glucose estimation, modeling of continuous glucose monitoring sensor drift and random error, and glucose prediction in diabetic patients (Zhou et al., 2018). By calculating the coefficients between adjacent mini-cycles, it is possible to assess whether each exercise cycle enhances or diminishes outcomes. In regression coefficients, the coefficients between cycles also demonstrated the same pattern. The correlation coefficient of Group 1 from T3 to T4 suddenly increased, indicating that the amount of blood glucose reduction in the subjects suddenly increased between the third and fourth periods. The results of the time for blood glucose improvement were the same as those shown by dawn phenomenon. The observed pattern in glucose response after each mini-cycle is analogous to the excess post-exercise oxygen consumption seen in aerobic and high-intensity interval training, where it reflects the body's delayed return to homeostasis (Sindorf et al., 2021). In our study, the changes in dawn phenomenon for Group 2 and Group 3 were relatively small across cycles, suggesting that although exercise induced fluctuations, blood glucose levels remained largely in fluctuation equilibrium. This may explain why, by the 9 mini-cycle, dawn phenomenon values were similar among all three intervention groups.

This study has several limitations. As a single-center trial, its generalizability is limited. Lifestyle factors were controlled only through verbal guidance, lacking objective monitoring and potentially introducing bias. Medication adjustments during the study, though recorded, could not be fully standardized. Moreover, the 9-cycle intervention represents a relatively short duration. Future multicenter, long-term studies with stricter medication control and objective lifestyle monitoring are warranted to validate these findings.

## 5 Conclusions

Periodic exercise significantly improved metabolic outcomes in patients with the dawn phenomenon, including enhancements in lipid metabolism, glucose metabolism, and a clear improvement in dawn phenomenon. Overall, the group with 1-day exercise rest days demonstrated greater improvements compared to the 2-day and 3-day interval groups.

## CRediT authorship contribution statement

**Yibo Gao:** Writing – review & editing, Writing – original draft, Software, Investigation, Formal analysis, Conceptualization. **Xiang Pan:** Writing – review & editing, Writing – original draft, Software, Formal analysis, Data curation. **Yanfeng Zhang:** Writing – review & editing, Visualization, Supervision, Resources, Project administration, Funding acquisition. **Koya Suzuki:** Writing – review & editing, Supervision, Methodology. **Mingzhe Li:** Supervision, Software, Methodology, Investigation. **Jin He:** Methodology, Investigation, Formal analysis. **Lupei Jiang:** Writing – review & editing, Supervision, Software, Investigation. **Jie Li:** Writing – review & editing, Supervision, Software, Resources, Investigation.

## Consent for publication

Not Applicable.

## Ethics approval and consent to participate

Approval was obtained from the Ethics Committee of the China Institute of Sports Science (No. CISSLA-20221116) and all participants provided written informed consent.

## Funding

This study was funded by 10.13039/501100002855Ministry of Science and Technology of the People's Republic of China (No. 2022YFC3600204), the Institute of Health and Sports Science & Medicine, 10.13039/501100005731Juntendo University, and the Research Encouragement Program of Juntendo University, Faculty of Health and Sports Science.

## Declaration of competing interest

The authors declare that they have no known competing financial interests or personal relationships that could have appeared to influence the work reported in this paper.

## Data Availability

Data will be made available on request.

## Appendix A Supplementary data

Supplementary material. Image 1
