# Supplementary material for: The role of combined training timing strategies in mitigating the dawn phenomenon in older adults with type 2 diabetes in Nanjing, China: a randomized controlled trial
Source: Prev Med Rep. 2026 Mar 21;65:103456. doi: 10.1016/j.pmedr.2026.103456 (PMC13049523; doi:10.1016/j.pmedr.2026.103456)
Supplement: Supplementary file 1 — Supplementary material. [file mmc1.docx]

Supplementary Materials

# Detailed Protocol

The exercise intervention protocol was modified based on the exercise prescription jointly promoted by American College of Sports Medicine and American Diabetes Association, and strictly designed according to the FITT-VP principles outlined in the exercise prescription.

During the 3-day adaptation period, participants first received instruction on exercise techniques (Day 1). On Day 2, intensity and the talk test were conducted. For safety reasons, given that the participants were elderly patients, intensity testing was performed using a 5-repetition maximum, from which the one-repetition maximum was estimated. A nurse assisted with instruction and measurements during the talk test. On Day 3, participants completed the formal adaptation period and the talk test at 70% of the prescribed training intensity.

Bodyweight Squat: Resistance was progressively increased in 2.5 kg increments (using dumbbells) based on the participant’s technique and performance, until five repetitions could no longer be completed.

Incline Wall Push: Resistance was initially set at 10 lb (using a resistance band) and increased in 5 lb increments according to the participant’s performance and movement quality, until five repetitions could no longer be completed.

Supine Leg Raise: Resistance started at 0.6 kg (using a sandbag) and was increased in 0.5 kg increments based on the participant’s technique and performance, until five repetitions could no longer be completed.

Each session lasted 90 minutes, including warm-up, inter-component rest days, and post-exercise stretching. Aerobic exercise was performed at a moderate intensity, defined as 40%–59% of the maximal heart rate, while resistance training was set at 50%–69% of one-repetition maximum.

According to previous research, the insulin-sensitizing effect of a single 60-minute moderate-intensity exercise session can be reversed within 48 hours. In addition, based on the principle of supercompensation, the intervention was structured as a 5-day mini-cycle, with training on Days 1, 3, and 5, and 24-hour rest (1 day) rest days in between. The interval between mini-cycles varied by group: 24 hours in Group 1, 48 hours (2 days) in Group 2, and 72 hours (3 days) in Group 3. The control group did not receive any exercise intervention . All three intervention groups completed nine mini-cycles (a total of 27 sessions), thereby ensuring equivalent total exercise volume across groups.


## 1.1 Exercise intervention

### 1.1.1 Frequency

The exercise prescription recommends engaging in aerobic exercise for 3–7 days per week and resistance training for 2–3 days per week on non-consecutive days. Based on the principle of exceeding compensation, the exercise program in this study was structured into 5-day cycles: participants exercised on the 1st, 3rd, and 5th days, with rest on the 2nd and 4th days. Group 1 had a 1-day interval between each cycle; Group 2 had a 2-day interval; Group 3 had a 3-day interval; Group 4 received no exercise intervention. All three exercise groups complied with the prescription requirements—namely, aerobic exercise performed 3–7 days per week with no more than 2 consecutive rest days, and resistance training conducted 2–3 days per week on non-consecutive days. A total of nine training cycles were completed. The exercise schedule is illustrated in Figure S1.


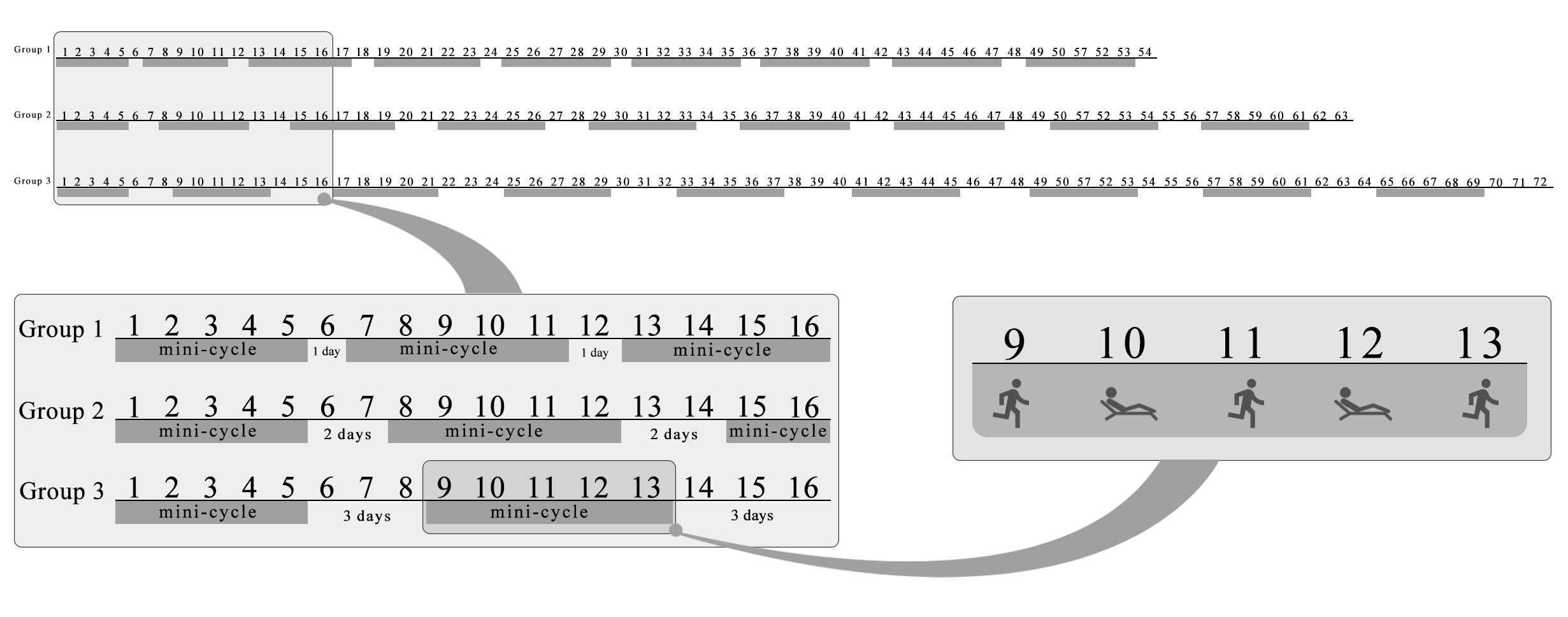


**Figure S1.** Schematic illustration of the periodic exercise schedule for three intervention groups among Chinese older adults with type 2 diabetes in Nanjing, China, from October 2023 to January 2024.

Note: 1 day, 2 days, and 3 days rest days refer to the rest periods between mini-cycles.

### 1.1.2 Intensity

The participants recruited for this study were all middle-aged and elderly individuals; therefore, moderate intensity was selected for both aerobic and resistance training. The moderate-intensity standard for aerobic exercise in the prescription was 40%–59% of maximal heart rate or rating of perceived exertion 10–13, while the moderate-intensity standard for resistance training was 50%–69% of one-repetition maximum. For aerobic exercise, maximal heart rate was used for intensity monitoring. In this study, all exercise protocols utilized bodyweight. Before starting the exercise, one-repetition maximum was first tested, and subsequently, during the exercise process, the moderate intensity standard was 50%–69% of one-repetition maximum. During warm-up and stretching, the intensity was chosen to the point of tightness or slight discomfort. Intensity monitoring used the rating of perceived exertion scale, with an rating of perceived exertion range of 10–13.

### 1.1.3 Time

In terms of exercise duration, the protocol followed the American College of Sports Medicine and American Diabetes Association recommendations for moderate-intensity physical activity. Aerobic exercise was scheduled for 150–300 minutes per week, while resistance training involved 1–3 sets of each exercise, with 10–15 repetitions per set. Each training day within a cycle included the following plan: from 9:00 to 10:30 a.m., participants performed a 10-minute warm-up, followed by 45 minutes of aerobic exercise consisting of 10–12-minute sessions with 3–5-minute breaks between them. Resistance training followed the aerobic session and consisted of three exercises, each performed for 2 sets of 10 repetitions, with 3-minute rests between exercises and a 5-minute rest between sets. After the resistance training, a 15-minute stretching session was conducted. During the entire session, the Rating of Perceived Exertion scale was used to monitor exercise intensity. Rating of perceived exertion ratings were collected at the end of every 10 minutes. If a participant's rating of perceived exertion exceeded 15, the session was terminated immediately to prevent potential injury. Additionally, heart rate was monitored and maintained within 40%–59% of maximal heart rate. If participants approached the upper threshold, the session was also stopped to ensure safety.

### 1.1.4 Type

The warm-up routine followed a sequential activation pattern starting from the ankles, calves, thighs, abdomen, back, shoulders, and finally the neck. This progression of small movements aimed to reduce muscular viscosity and minimize the risk of injury. Due to limitations in the training space, aerobic exercise was conducted in the form of aerobic gymnastics. A total of 10 movements were designed, including 1 foot movement, 2 leg movements, 4 waist movements, 2 shoulder movements, and 1 neck movement. Detailed descriptions of the movements are provided in Table S1. The resistance training component consisted of 3 exercises: one targeting the lower limbs, one for the upper limbs, and one for the trunk. Specific safety considerations for each exercise are outlined in Table S2. The cool-down phase involved static stretching, following the same order as the warm-up: feet, calves, thighs, abdomen, back, shoulders, and neck.

**Table S1.** Types and Movements Standards of combined aerobic and resistance training for Chinese older adults with type 2 diabetes in Nanjing, China (October 2023 to January 2024).

| **Aerobic Exercise Movements Types** | **Standards** |
| --- | --- |
| Lunge Foot Press | ①Stand with feet shoulder-width apart.  ②During the movement, keep the outer edge of the foot in contact with the ground while turning the sole inward as much as possible. Place hands on hips or extend arms sideways for balance.  ③Each repetition counts when returning to the starting position after one downward press. |
| Forward Lunge with Torso Extension | ①Step forward with the left foot into a lunge position.  ②Raise both arms from a lowered position to an overhead stretch while extending the back from a bent position to an upright or slightly arched posture, drawing the arms backward as much as possible.  ③Maintain the lunge position throughout the movement; Each repetition is counted from the upward to downward motion. Perform 5 repetitions, then switch legs and repeat with the right foot forward. |
| Side Lunge with Torso Extension | ①Begin in a lunge stance, then move the foot and leg 45 degrees toward the same side to form a side lunge. Perform the same upper-body movements and repetition count as in the forward lunge with torso extension. ②Ensure that the torso and face remain aligned with the direction of the bent knee. |
| Cross-Step Lunge with Torso Extension | ①Step into a wide cross-lunge position within your range of ability.  ②Perform the same movements and repetition pattern as in the forward lunge with torso extension.  ③Follow the same posture and alignment precautions as in the side lunge. |
| Forward Lunge with Knee Flexion | ①The leg position is the same as in the forward lunge with torso extension.  ②Keep the back straight throughout the movement.  ③Flex the front knee from an upright to a bent position while maintaining arm balance. Perform 5 repetitions on each leg. |
| Side Lunge with Knee Flexion | ①The leg movement mirrors that of the side lunge with torso extension.  ②The movement pattern is the same as the forward lunge with knee flexion.  ③Keep the torso and face aligned with the direction of the bent knee. Perform 5 repetitions on each side. |
| Trunk Rotation | ①Stand with feet shoulder-width apart; bend both arms and hold them in front of the chest.  ②Rotate the torso to the left and right within a comfortable range of motion. Perform 10 repetitions on each side. |
| Lunge with Arm Swing | ①Assume a forward lunge stance.  ②Raise the opposite arm upward and backward toward the outside.  ③Swing the arm in a curved motion from in front of the body toward the foot on the lunging side.  ④To avoid orthostatic hypotension, movements should be slow and controlled.  ⑤Perform 10 repetitions on each side per set. |
| Shoulder Rotation | ①Stand with feet shoulder-width apart; extend both arms sideways, perpendicular to the torso.  ②Make large forward circles with both arms 10 times; then reduce the circle size and repeat 10 times; then reduce further and complete another 10 repetitions.  ③Rest for 30 seconds and repeat the same process in the reverse direction (backward). |
| Neck Movement | ①Stand with feet shoulder-width apart. Move the head gently in the following directions: forward, backward, left, and right; Perform 5 repetitions in each direction.  ②Movements should be slow and controlled. |
| **Resistance Training Movements** **Types** | **Standards** |
| Bodyweight Squat | ①Stand upright with your head raised and chest lifted; place both hands behind the head.  ②Draw in the abdomen (engage the core by pulling the navel inward).  ③Lower the body into a half-squat position, maintaining proper alignment to avoid compensation: knees should track in line with the toes, and feet should point forward.  ④Extend the hips, knees, and ankles to return to the standing position. Repeat. |
| Incline Wall Push | ①Stand with the upper body upright, both arms extended to touch the wall. Position one leg forward (knee bent) and the other leg extended straight back with the heel touching the ground.  ②Perform 12 wall pushes: press the wall with both hands while simultaneously contracting the abdominal and gluteal muscles; keep the rear leg fully extended at the knee and ankle.  ③Do not allow the heel of the back foot to lift during the push. Extend the elbows and contract the chest muscles to return to the starting position. Ensure the head remains aligned (no forward lean). |
| Supine Leg Raise | ①Lie flat on the floor in a supine position.  ②Keep both legs straight and together; slowly raise them to 15–20 cm above the floor. Hold the position for 2–3 seconds.  ③Slowly lower the legs back down while keeping them extended.  ④Exhale during the upward motion and inhale on the way down. One full raise and lower counts as one repetition. |

### 1.1.5 Total Volume of Exercise

This study was designed based on the exercise recommendations provided by American College of Sports Medicine and American Diabetes Association regarding the frequency, intensity, time, type, and volume of exercise for diabetic patients. Literature reviews support that a combination of aerobic and resistance training yields optimal outcomes. Accordingly, the exercise prescription in this study included: Aerobic exercise: 150–300 minutes per week of moderate-intensity activity or 75–150 minutes of vigorous-intensity activity, performed 3–7 days per week, with no more than 2 consecutive days without exercise. Resistance training: Exercises targeting the upper limbs, trunk, and lower limbs, with 10–15 repetitions per set, 1–3 sets per movement, and 2–3 non-consecutive sessions per week. To meet these guidelines and accommodate middle-aged and elderly participants, the program adopted a 5-day cycle as the basic unit. Each cycle was followed by a rest interval tailored to different intervention groups. A total of 9 cycles were implemented and divided into three progressive phases to allow for adaptation. Within each 5-day cycle, the total training volume was approximately: Moderate-intensity aerobic exercise: 165–200 minutes; Moderate-intensity resistance training: 60–120 minutes. This structure ensured that the overall exercise load aligned with clinical recommendations for moderate-intensity training in individuals with diabetes.

### 1.1.6 Progression

The intervention began with a 3-day familiarization period to allow participants to experience the initial exercise intensity, followed by a 1-day adaptation phase before formal training commenced. The progression of the protocol was individualized and adjusted based on each participant's completion time per exercise, total duration, and baseline characteristics such as age, physical fitness, health status, and personal goals. For resistance training, the number of sets was increased progressively within participants' tolerance levels. Stretching intensity and range were also gradually expanded over time.

1. **Participant Screening and Eligibility**

The study included older adults aged between 60.00 and 75.00 years who were diagnosed with the dawn phenomenon (defined as a rise ≥ 1.11 mmol/L). Eligible participants were required to be willing to provide informed consent, demonstrate normal exercise capacity, and have had no initiation or adjustment of glucose-lowering or lipid-lowering medications or structured exercise programs within the 2.00 weeks prior to enrollment. Furthermore, participants must have maintained stable sleep habits for at least 2.00 weeks.

Exclusion criteria were as follows: (1) diagnosis of type 1 diabetes or gestational diabetes; (2) evidence of the Somogyi phenomenon, defined as nocturnal hypoglycemia (< 3.90 mmol/L between 00:00 and 03:00 AM) followed by rebound fasting hyperglycemia (> 7.00 mmol/L); (3) presence of serious complications such as myocardial infarction, stroke, end-stage renal disease, or proliferative retinopathy, or any hospitalization within the past 3.00 months; (4) initiation or adjustment of insulin, GLP-1 receptor agonists, or SGLT2 inhibitors within the past 3.00 months.

# Dietary Control Protocol

Before the intervention, participants' basal metabolic rate was estimated based on physical examination results, and daily exercise-related energy expenditure was calculated using cardiopulmonary exercise testing data. Personalized nutritional recommendations were then provided. The dietary plan ensured adequate caloric intake to support daily energy needs, with distribution as follows: Protein: 15%–25% of total daily calories, emphasizing high-quality sources to maintain nitrogen balance during aerobic exercise. Fat: Strictly controlled, accounting for 15%–25% of total intake. Carbohydrates: 50%–70% of total daily calories. Participants received guidance on this dietary structure, and adherence was monitored through questionnaires to minimize the confounding effects of diet on blood glucose levels.

# Experimental Procedure

Twenty-four hours after the exercise familiarization session, fasting participants underwent baseline biochemical and physical fitness assessments. Following data collection, a continuous glucose monitoring system was applied, and participants were provided with fingertip blood sampling kits. After confirming continuous glucose monitoring stability with an initial fingertip blood samples cross-check, subsequent fingertip blood samples were self-collected and submitted daily via screenshots in a designated WeChat group. Participants were informed of the continuous glucose monitoring sensor replacement schedule. The formal exercise intervention was conducted across seven community venues, with exercise sessions organized by group-specific schedules. Each group followed its assigned training plan, and continuous glucose monitoring data were collected 24 hours after the end of each exercise cycle. Community workers led and supervised each session, ensuring attendance and compliance via check-ins before exercise. Exercise intensity was monitored using the rating of perceived exertion scale. All groups completed nine intervention cycles, and post-intervention indicators were reassessed 24 hours after the final session (Figure S2).


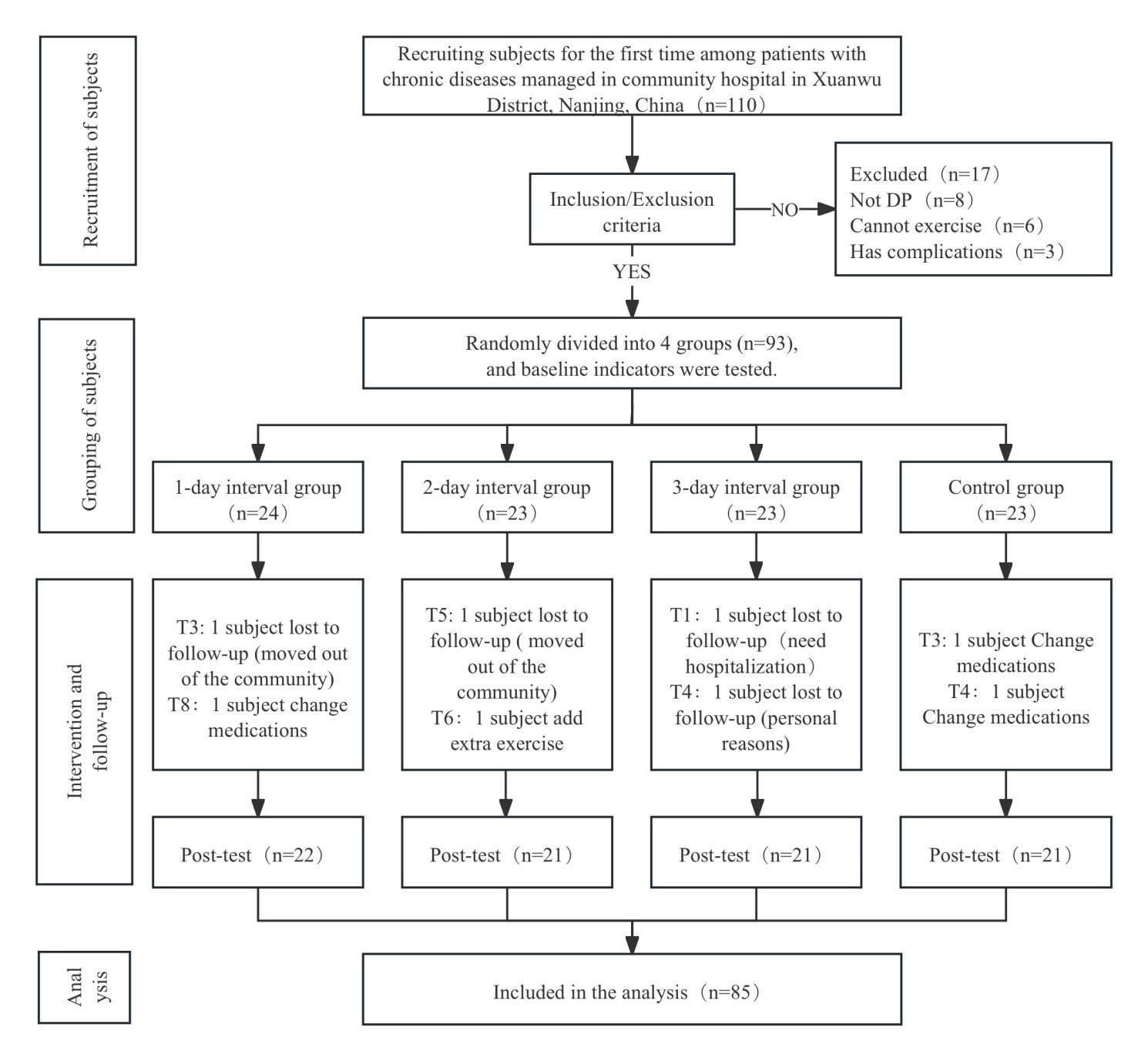


**Figure S2.** CONSORT flow diagram of participant screening, randomization, and analysis among Chinese older adults with type 2 diabetes in Nanjing, China, from October 2023 to January 2024.

T1, T2......T9 represent the 1st and 2nd......9 Mini-cycles, respectively.

# Equipment calibration

We utilized the Yuwell 310 fingertip blood glucose device to collect fingertip blood glucose readings (fasting, 2 hours after breakfast, and 2 hours after dinner) for calibrating the continuous glucose monitoring. We employed the first round of wear data (N=1012) to assess calibration accuracy using the Mean Absolute Relative Deviation. Additionally, we calculated the standard deviation using the Bland-Altman method within MedClac 23.3.4 software. Results indicated a Mean Absolute Relative Deviation of 8.43% and a standard deviation of 0.05 mmol/L (95% CI, [0.01, 0.01]), with 95% limits of agreement ranging from –1.85 to 1.76 mmol/L, confirming that continuous glucose monitoring values were consistent with capillary glucose.

# **6.** Subject medication status

**Table S2.** Medication adherence status among Chinese older adults with type 2 diabetes mellitus during the intervention period in Nanjing, China (October 2023 to January 2024).

| Type | Subjects | 3 times per day | 2 times per day | 1 time per day |
| --- | --- | --- | --- | --- |
| Biguanides | 39 | 26 | 10 | 3 |
| Sulfonylureas | 19 | 15 | 4 | 0 |
| α-Glucosidase inhibitors | 7 | 4 | 3 | 0 |
| Dipeptidyl peptidase-4 (DPP-4) inhibitors | 8 | 6 | 2 | 0 |
| Glinides | 12 | 8 | 3 | 1 |

**Table S3.**Detailed mean differences, standard errors, 95% confidence intervals, and effect sizes for metabolic indicators among Chinese older adults with type 2 diabetes mellitus in Nanjing, China, from October 2023 to January 2024. (mmol/L)

| Indicators | Group | Change in Mean (Absolute Value) | Change in SEM (Absolute Value) | Mean Different | 95%CI | Effect Size(Conhen,s d) |
| --- | --- | --- | --- | --- | --- | --- |
| Total cholesterol (mmol/L) | 1B-1P | 16.90 | 3.60 | 0.41 | [0.25, 0.63] | 1.04 |
|  | 2B-2P | 20.03 | 4.17 | 0.53 | [0.29, 0.75] | 1.04 |
|  | 3B-3P | 26.32 | 5.74 | 0.74 | [0.37, 0.99] | 0.99 |
| Triglycerides (mmol/L) | 1B-1P | 0.61 | 0.13 | 0.62 | [0.33, 0.87] | 0.98 |
|  | 2B-2P | 0.43 | 0.08 | 0.38 | [0.23, 0.57] | 1.08 |
|  | 3B-3P | 0.37 | 0.09 | 0.41 | [0.21, 0.59] | 0.98 |
| High-density lipoprotein (mmol/L) | 1B-1P | 0.56 | 0.13 | 0.59 | [0.33, 0.87] | 0.98 |
|  | 2B-2P | 0.51 | 0.10 | 0.48 | [0.29, 0.71] | 1.09 |
|  | 3B-3P | 0.49 | 0.11 | 0.53 | [0.27, 0.73] | 1.00 |
| Low-density lipoprotein(mmol/L) | 1B-1P | 0.53 | 0.11 | 0.51 | [0.27, 0.73] | 0.96 |
|  | 2B-2P | 0.31 | 0.06 | 0.32 | [0.17, 0.43] | 1.11 |
|  | 3B-3P | 0.51 | 0.11 | 0.51 | [0.27, 0.73] | 1.00 |
| Indicators | Group | Pre-test (Mean ± SD) | Post-test (Mean ± SD) | Mean Change | 95% CI of Change | Effect Size (Cohen's d) |
| Lipid Profiles |  |  |  |  |  |  |
| Total cholesterol (mmol/L) | Group 1 | 4.91 ± 0.81 | 4.02 ± 0.85 | 0.89 | [-1.14, -0.66] | 1.05 |
|  | Group 2 | 5.00 ± 1.22 | 4.48 ± 1.28 | 0.52 | [-0.85, -0.15] | 0.41 |
|  | Group 3 | 4.83 ± 1.04 | 4.33 ± 0.81 | 0.50 | [-0.86, -0.34] | 0.68 |
| Triglycerides (mmol/L) | Group 1 | 1.71 ± 1.03 | 1.14 ± 0.72 | 0.57 | [-0.87, -0.33] | 0.66 |
|  | Group 2 | 1.69 ± 0.89 | 1.31 ± 0.67 | 0.38 | [-0.57, -0.23] | 0.53 |
|  | Group 3 | 1.61 ± 1.02 | 1.22 ± 0.91 | 0.39 | [-0.59, -0.21] | 0.43 |
| High-density lipoprotein (mmol/L) | Group 1 | 1.40± 0.31 | 1.87 ± 0.19 | -0.47 | [+0.27, +0.73] | 1.80 |
|  | Group 2 | 1.38 ± 0.36 | 1.69 ± 0.40 | -0.31 | [+0.17, +0.43] | 0.75 |
|  | Group 3 | 1.29 ± 0.28 | 1.72 ± 0.24 | -0.43 | [+0.27, +0.73] | 2.01 |
| Low-density lipoprotein(mmol/L) | Group 1 | 2.86 ± 0.83 | 2.27 ± 0.76 | 0.59 | [-0.87, -0.33] | 0.77 |
|  | Group 2 | 3.00 ± 0.81 | 2.49 ± 0.67 | 0.51 | [-0.71, -0.29] | 0.68 |
|  | Group 3 | 3.01 ± 0.78 | 2.51 ± 0.60 | 0.50 | [-0.73, -0.27] | 0.71 |
| Glucose Metabolism |  |  |  |  |  |  |
| Fasting blood glucose (mmol/L) | Group 1 | 7.92 ± 1.13 | 6.11 ± 0.91 | 1.81 | [-2.33, -1.27] | 1.84 |
|  | Group 2 | 8.29 ± 1.76 | 7.12 ± 1.21 | 1.17 | [-1.98, -0.42] | 0.79 |
|  | Group 3 | 8.31 ± 1.77 | 6.61 ± 1.18 | 1.70 | [-2.52, -0.88] | 1.10 |
| Hemoglobin A1c (%) | Group 1 | 7.10 ± 1.33 | 6.14 ± 1.16 | 0.96 | [-1.55, -0.45] | 0.79 |
|  | Group 2 | 7.46 ± 1.37 | 6.36 ± 1.28 | 1.10 | [-1.69, -0.51] | 0.80 |
|  | Group 3 | 6.75 ± 1.29 | 6.17 ± 1.17 | 0.58 | [-1.13, -0.07] | 0.48 |
| Dawn phenomenon(mmol/L) | Group 1 | 2.39 ± 1.11 | 0.51 ± 0.84 | 1.88 | [-2.43, -1.37] | 1.96 |
|  | Group 2 | 2.21 ± 0.82 | 0.62 ± 0.88 | 1.59 | [-2.05, -1.15] | 1.88 |
|  | Group 3 | 2.42 ± 1.19 | 0.61 ± 1.11 | 1.81 | [-2.41, -1.19] | 1.58 |


Note: SD, standard deviation; CI, confidence interval. Effect size is represented by Cohen's d. According to standard guidelines, Cohen's d values of 0.2, 0.5, and 0.8 are considered to represent small, medium, and large effect sizes, respectively.1B is the Group 1 baseline test and 1P is the Group 1 post test; 2B is the Group 2 baseline test and 2P is the Group 2 post test; 3B is the Group 3 baseline test and 3P is the Group 3 post test.

**Table S4.** Goodness-of-fit indices for the autoregressive models across the three intervention groups among Chinese older adults with type 2 diabetes mellitus in Nanjing, China, from October 2023 to January 2024.

| **Group** | **chi-square divided by degrees of freedom** | **Root Mean Square Error of Approximation,** | **Goodness of Fit Index** | **Comparative Fit Index** | **Incremental Fit Index** |
| --- | --- | --- | --- | --- | --- |
| Recommended Guidelines | < 5.00 | 0.08 | > 0.80 | > 0.80 | > 0.80 |
| Group 1 | 1.09 | 0.04 | 0.87 | 0.92 | 0.93 |
| Group 2 | 2.36 | 0.06 | 0.86 | 0.91 | 0.92 |
| Group 3 | 2.62 | 0.06 | 0.87 | 0.93 | 0.88 |
